# *In vivo* effects of bempedoic acid on microdosed CYP probe drugs

**DOI:** 10.3389/fphar.2025.1544956

**Published:** 2025-04-08

**Authors:** Felicitas Stoll, Salvatore Amato, Jürgen Burhenne, Antje Blank

**Affiliations:** Internal Medicine IX - Department of Clinical Pharmacology and Pharmacoepidemiology, Heidelberg University, Medical Faculty Heidelberg/Heidelberg University Hospital, Heidelberg, Germany

**Keywords:** microdosed probe drugs, CYP3A, CYP2D6, CYP2C19, bempedoic acid, limited sampling strategy

## Abstract

**Background:**

Bempedoic acid (BA) is a novel oral cholesterol-lowering drug. So far, *in vivo* evidence on potential drug–drug interactions via the cytochrome P450 (CYP) enzymes is lacking.

**Methods:**

In a clinical trial, we evaluated the effect of BA on microdosed probe drugs using a limited sampling strategy in healthy volunteers. The outcome measures were as follows: 1) the omeprazole AUC_0–4h_ and hydroxylation index (HI) after a 100 µg dose to evaluate CYP2C19 activity, 2) the midazolam AUC_2–4h_ after a 30 µg dose to evaluate CYP3A activity, and 3) the yohimbine AUC_0–4h_ after a 50 µg dose to evaluate CYP2D6 activity. Partial areas under the curve (AUCs) were evaluated at baseline and under BA steady state. The endpoints were the geometric mean ratios (GMRs) with 95% confidence intervals (CI) of the partial AUCs.

**Results:**

In 15 participants, the AUC_0–4h_ of omeprazole and its HI significantly decreased (GMR: 0.75, 90% CI: 0.66–0.85; change in HI *p* < 0.0001). There was no change in the AUC_2–4h_ of midazolam (GMR: 1.18, 90% CI: 0.87–1.61) and AUC_0–4h_ of yohimbine (GMR: 0.92, 90% CI: 0.75–1.14).

**Conclusion:**

In healthy volunteers, BA was a mild inducer of CYP2C19 and did not affect CYP3A or CYP2D6 activity.

## 1 Introduction

Bempedoic acid (BA) is a novel oral cholesterol-lowering drug that acts by inhibiting adenosine triphosphate citrate lyase, an enzyme involved in the hepatic cholesterol synthesis; this inhibition occurs upstream of 3-hydroxy-3-methyl-glutaryl-coenzyme A reductase, which is targeted by the statins (EMA, 2024). Its positive effects on LDL cholesterol (LDL-C) and cardiovascular endpoints in patients with increased cardiovascular risk—including those with full or partial statin intolerance—have been demonstrated in large clinical trials (Nissen et al., 2023; Ray et al., 2019). BA can be administered in conjunction with statin therapy to achieve evidence-based LDL-C goals and as a monotherapy or in combination with other cholesterol-lowering drugs for patients who are statin-intolerant. It is available as a single-substance drug or as a combination pill with ezetimibe (EMA, 2024; EMA, 2020). Thus, it is a relevant new option to combine these oral drugs for the treatment of hypercholesterolemia.

The pharmacokinetics of BA have been described in detail elsewhere by us and other authors (Cicero et al., 2021; Stoll et al., 2022; Stoll et al., 2025; Amore et al., 2023). Importantly, BA is administered as a prodrug that is activated at the site of action; this is expected to reduce the risk for off-site adverse effects, particularly those related to muscle. After absorption, the maximum concentration is reached after 3.5 h. The volume of distribution is 18 L, and plasma binding is high (99%) (EMA, 2024; Cicero et al., 2021). BA has an active metabolite, ESP15228, with a plasma exposure of approximately 20% that of the parent substance (Stoll et al., 2025; Amore et al., 2023). Both are inactivated by glucuronidation and are mainly excreted in urine (70%) (EMA, 2024; Amore et al., 2023). The steady-state half-life of BA is 19 h (EMA, 2024).

*In vitro* and clinical interaction studies revealed that BA inhibits the hepatic transporter OATP1B1 (EMA, 2024). In contrast, *in vitro* studies indicate that BA has no relevant effect on the cytochrome P450 (CYP) enzymes. Specifically, there was no significant direct inhibition of CYP1A2, CYP2A6, CYP2B6, CYP2C8, CYP2C9, CYP2C19, CYP2D6, CYP2E1, or CYP3A4 with BA concentrations up to approximately 100 μg/mL; furthermore, IC_50_ values for these CYP isoenzymes did not change significantly after pre-incubation with BA, making time-dependent inhibition unlikely. Regarding the *in vitro* induction of CYP enzymes, there was no inducing effect on CYP1A2 or CYP2B6 enzyme activity. BA induced the activities of CYP2C8, CYP2C9, CYP2C19, and CYP3A4 at a high BA concentration of approximately 100 μg/mL. However, because this concentration is well above the human exposure at steady state (20 μg/mL), *in vivo* induction was deemed unlikely (FDA, 2019).

However, *in vivo* data on the interaction with CYP isoenzymes are lacking. The use of microdosed probe drugs is well-established for the phenotyping of CYP enzymes (van der Heijden et al., 2024), and we have proven the value of drug microdosing in many projects (Burhenne et al., 2012; Halama et al., 2013; Mahmoudi et al., 2021; Muhareb et al., 2023; Rohr et al., 2024; Stoll et al., 2024; Vay et al., 2020; Vay et al., 2019; Elbe et al., 2022). A significant advantage of this approach is that microdosed probe drugs, unlike standard doses, do not produce therapeutic effects while maintaining CYP specificity. We investigated the effect of BA on CYP3A, CYP2D6, and CYP2C19 using midazolam (Halama et al., 2013; Rohr et al., 2024; Stoll et al., 2024), yohimbine (Stoll et al., 2024; Vay et al., 2020), and omeprazole (Muhareb et al., 2023; Rohr et al., 2024; Elbe et al., 2022) as microdosed probe drugs.

## 2 Participants and methods

### 2.1 Trial conduct

The trial design is described in detail by Stoll et al. (2025). In brief, we conducted an open-label phase I drug interaction trial in healthy volunteers at the ISO-certified Pharmacological Early Clinical Trial Center (KliPS) at Heidelberg University Hospital, Department of Clinical Pharmacology and Pharmacoepidemiology (Internal Medicine IX), from July 2022 to June 2023. The trial was approved by the national authority and the responsible Ethics Committee of the Medical Faculty of Heidelberg University (27 June 2022, AFmo-366/2022), and it was conducted according to the guidelines of Good Clinical Practice, the ethical principles expressed in the Declaration of Helsinki, and all legal requirements for clinical trials in Germany and the European Union. The fully informed participants provided their written consent before any trial procedures.

Participants included were aged 18–45 years and in full physical and mental health. The intake of medications (with the exception of iodine, thyroxine, and oral contraception) was not allowed during the trial. Furthermore, all substances (e.g., food supplements) known to inhibit or induce CYP enzymes, grapefruit products, and cannabis were prohibited.

The influence of BA on CYP2D6, CYP2C19, and CYP3A4 was assessed through microdose phenotyping at BA steady state, conducted on at least the 5th day of BA intake (optional +2 days) in the evening (standard dose of 180 mg daily; Nilemdo Filmtabletten, Daiichi Sankyo Europe). This was an independent secondary endpoint. The primary objective was to evaluate the interaction between BA and pravastatin, which is reported separately (Stoll et al., 2025).

### 2.2 Microdosed trial medication and blood sampling

For the evaluation of CYP3A activity, the area under the curve (AUC) or the clearance of microdosed midazolam is an established phenotyping strategy, with experience spanning doses from 0.1 to 75 µg (van der Heijden et al., 2024; Burhenne et al., 2012; Halama et al., 2013; Muhareb et al., 2023; Rohr et al., 2024; Stoll et al., 2024). CYP2C19 activity can be phenotyped using the AUC of microdosed omeprazole at a dose of 100 µg (van der Heijden et al., 2024; Mahmoudi et al., 2021; Muhareb et al., 2023; Rohr et al., 2024; Elbe et al., 2022). Similarly, CYP2D6 activity can be determined using the AUC of microdosed yohimbine at a dose of 50 µg (van der Heijden et al., 2024; Rohr et al., 2024; Stoll et al., 2024; Vay et al., 2020; Vay et al., 2019).

The microdosed drugs were administered as follows: midazolam as an oral solution at a dose of 30 µg (Dormicum V^®^ 5 mg/5 mL Lösung, Roche Pharma AG) in 50 mL of water; yohimbine at a dose of 50 µg as tablets (Yohimbinum hydrochloricum D4 DHU Tabletten, DHU-Arzneimittel GmbH & Co. KG); and omeprazole at a dose of 100 µg as an oral solution (OMEP^®^ 40 mg, powder for the solution for infusion, HEXAL AG) in 100 mL of 4.2% sodium bicarbonate (Natriumhydrogencarbonat 4.2% B. Braun Infusionslösung) to prevent the gastric degradation of omeprazole.

All microdosed drugs (along with BA) were taken together. Ten minutes prior to the intake of medication, 50 mL of sodium bicarbonate (4.2%) was administered to buffer gastric acid.

The participants fasted (water was allowed) at least 6 h prior to the administration and 4 h thereafter.

Blood samples were collected in lithium heparin tubes. A limited sampling strategy (Katzenmaier et al., 2010) was undertaken with the following schedules: midazolam: predose, 2, 2.5, 3, and 4 h; yohimbine and omeprazole: predose, 0.5, 1, 1.5, 2, 2.5, 3, and 4 h.

### 2.3 Quantification of CYP3A, CYP2D6, and CYP2C19 probe drugs

The plasma quantification of midazolam/1-OH-midazolam (Burhenne et al., 2012), yohimbine (Vay et al., 2019), and omeprazole/5-OH-omeprazole (Mahmoudi et al., 2021; Elbe et al., 2022) was performed using the already published UPLC–MS/MS methods. The lower limit of quantification was 1 pg/mL for all compounds, and the quality control was performed strictly according to the ICH M10 guideline (ICH, 2022) under GCLP conditions.

### 2.4 Pharmacokinetic and statistical analysis

Outcome measures included the partial area under the concentration–time curve AUC_2–4h_ for midazolam and its metabolite 1-OH-midazolam derived from the concentration–time curve, the AUC_0–4h_ for yohimbine as well as for omeprazole and its metabolite 5-OH-omeprazole, and the hydroxylation index of omeprazole, defined as the molar ratio of omeprazole to 5-OH-omeprazole from the single 3 h-samples. Non-compartmental pharmacokinetic (PK) analysis (a standard pharmacokinetic assessment with one compartment) was performed using Phoenix WinNonlin Version 8.4 (Certara, Princeton, NJ, United States). PK data are presented as geometric means with 95% confidence intervals (CIs) and were compared using ratio-paired t-tests. The geometric mean ratios (GMRs) are shown with a 90% CI. The hydroxylation indices of omeprazole are presented as the arithmetic mean with a 95% CI and were compared using the Wilcoxon test. Demographic data are presented as the arithmetic mean ± standard deviation. The statistical analysis was conducted in GraphPad Prism Version 10 (La Jolla, CA, United States). A *p*-value <0.05 was considered significant.

## 3 Results

Fifteen participants (eight female individuals) were evaluated for microdose PK. They had a mean age of 26 y ± 4 and a body mass index of 23.3 kg/m^2^ ± 2.6.

**FIGURE 1:**
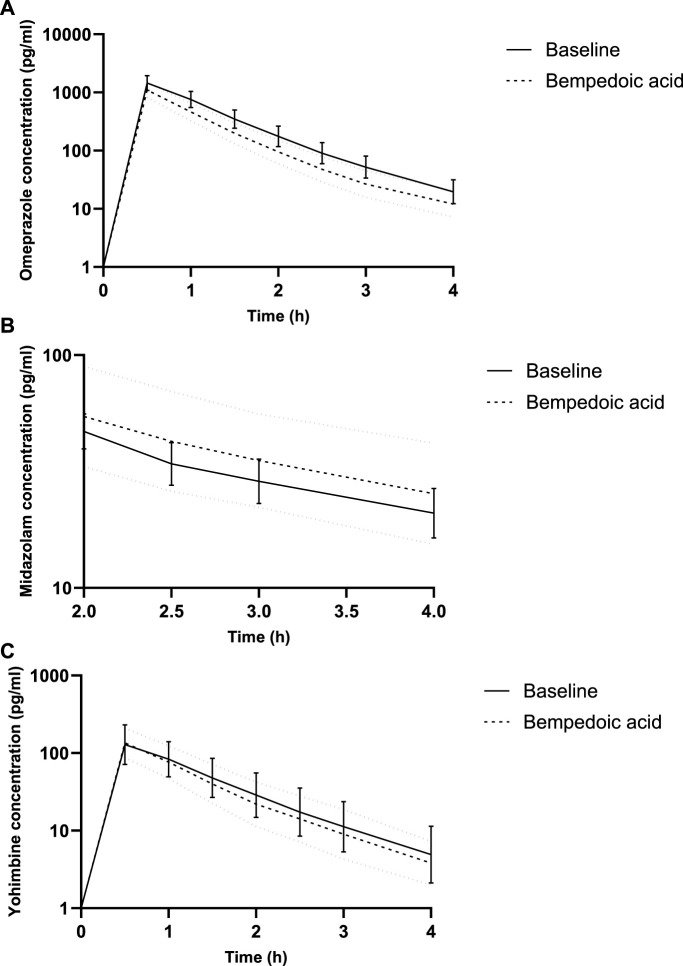
Concentration–time curves for microdosed omeprazole **(A)**, midazolam **(B)**, and yohimbine **(C)**, showing a limited sampling strategy. Geometric mean and 95% confidence interval.

Under BA, the partial AUC of omeprazole significantly decreased by 25%, indicating the induction of CYP2C19. Concurrently, the partial AUC of 5-OH-omeprazole increased by 17%. Furthermore, the hydroxylation index of omeprazole decreased significantly. The partial AUC of midazolam as a marker for CYP3A activity and that of yohimbine as a marker for CYP2D6 activity remained unchanged (Table 1; Figure 1).

**TABLE 1:** Pharmacokinetic parameters of microdosed cytochrome P450 probe drugs at baseline and with BA dosed to steady state in healthy volunteers (n = 15).

	Baseline		Bempedoic acid		Comparison		
	GM	95% CI	GM	95% CI	GMR	90% CI	*p*-value
Omeprazole							
AUC_0–4h_ (h * pg/mL)	1276	995–1636	955	688–1325	**0.75**	**0.66–0.85**	**0.0013**
5-OH-omeprazole							
AUC_0–4h_ (h * pg/mL)	1660	1484–1858	1945	1677–2255	**1.17**	**1.08–1.28**	**0.0057**
Hydroxylation index (HI)							
Molar ratio of omeprazole to 5-OH-omeprazole from single 3 h sample (mean)	0.4556	0.2370–0.6742	0.1517	0.09776–0.2057			**< 0.0001**

Midazolam							
AUC_2–4h_ (h * pg/mL)	62.4	49.3–78.9	73.7	45.5–119	1.18	0.87–1.61	0.36
1-OH-midazolam							
AUC_2–4h_ (h * pg/mL)	30.8	26.3–36.0	37.0	23.2–59.0	1.19	0.89–1.59	0.32

Yohimbine							
AUC_0–4h_ (h * pg/mL)	172	98.1–303	159	94.5–268	0.92	0.75–1.14	0.51

AUC, area under the plasma concentration–time curve; CI, confidence interval; GM, geometric mean; GMR, geometric mean ratio. Bold values are statistically significant.

## 4 Discussion

This pharmacokinetic evaluation with microdosed CYP probe drugs indicated that BA does not affect CYP3A and CYP2D6 activity but induces CYP2C19. The inducing effect of 25% is mild according to the definition of the U.S. Food and Drug Administration (FDA) (FDA, 2024). An inducing effect of BA on CYP2C19 was also found *in vitro* but was considered unlikely to be relevant for humans due to the expected plasma concentrations of BA (FDA, 2019). Intrahepatic concentrations–in addition to the plasma concentration of BA–might be of primary importance.

A significant number of patients receiving cholesterol-lowering drugs also take other medications, like platelet inhibitors, antihypertensives, beta-blockers, glucose-lowering drugs, or drugs for the treatment of peptic ulcer (Corn et al., 2024); these potential drug interactions should be considered. Concomitant treatment with statins or other OATP substrates must now be taken into account for potential drug interactions (EMA, 2024). Based on our results, cardiovascular and non-cardiovascular CYP2C19 substrates—including clopidogrel and neuropsychiatric drugs, such as amitriptyline, citalopram, and phenytoin, in addition to proton pump inhibitors (Asiimwe and Pirmohamed, 2022)—may need further investigation in the future.

It should be advised to monitor the clinical effects and, in selected cases, the drug levels of CYP2C19 substrates that are co-administered with BA to detect a lack of efficacy. The quantification of drug levels could be useful for CYP2C19 substrates with a narrow therapeutic range or/and a strong recommendation for therapeutic drug monitoring, e.g., amitriptyline or phenytoin (Hiemke et al., 2018). In the case of clopidogrel, the induction of CYP2C19 is expected to increase the level of the active metabolite, which could be associated with an increased risk of bleeding. The SmPC of clopidogrel advises against the concomitant use of strong CYP2C19 inducers (SmPC, 2022). The relevance of BA with only mild inducing effects in this study remains unclear.

Although our findings–based on a microdosing approach after a limited time of BA intake–are supported by previous *in vitro* results on CYP2C19 induction, it should be pointed out that the magnitude of the effect might differ with the long-term BA administration or full therapeutic doses of the affected drug.

We chose the microdosing approach because of its minimized risk of undesired drug effects. Although microdosing for CYP phenotyping is widely established, evidence differs across CYPs, with some limitations remaining (van der Heijden et al., 2024). We used a limited sampling strategy, as developed and applied in previous studies (Rohr et al., 2024; Stoll et al., 2024; Katzenmaier et al., 2010). The use of microdosed omeprazole necessitates the administration of sodium bicarbonate to buffer gastric acid. It cannot be excluded that this affects the absorption of the other CYP probe drugs, although this was found to be unlikely in previous studies (Rohr et al., 2024; Elbe et al., 2022).

CYP2D6 activity is highly variable depending on the genotype, thus leading to substantial interindividual variability in yohimbine partial AUC (Stoll et al., 2024; Vay et al., 2020). We cannot entirely rule out an effect of BA on CYP2D6 due to the sample size of our trial. However, BA showed no inhibitory effect on CYP2D6 *in vitro*, and CYP2D6 is not inducible; so taken all together, it is unlikely that BA interacts with CYP2D6 in a clinically relevant manner.

In conclusion, our results indicate that BA possesses a mild CYP2C19-inducing effect, while there was no detectable effect on CYP3A4 and CYP2D6.

## Data Availability

The raw data supporting the conclusions of this article will be made available by the authors, without undue reservation.

## References

[B1] Amore B. M. Cramer C. MacDougall D. Emery M. G. (2023). The disposition and metabolism of bempedoic acid, a potent inhibitor of ATP citrate lyase, in healthy human subjects. Drug Metab. Dispos. 51 (5), 599–609. 10.1124/dmd.122.001142 36878717

[B2] Asiimwe I. G. Pirmohamed M. (2022). Drug-drug-gene interactions in cardiovascular medicine. Pharmgenomics Pers. Med. 15, 879–911. 10.2147/PGPM.S338601 36353710 PMC9639705

[B3] Burhenne J. Halama B. Maurer M. Riedel K. D. Hohmann N. Mikus G. (2012). Quantification of femtomolar concentrations of the CYP3A substrate midazolam and its main metabolite 1'-hydroxymidazolam in human plasma using ultra performance liquid chromatography coupled to tandem mass spectrometry. Anal. Bioanal. Chem. 402 (7), 2439–2450. 10.1007/s00216-011-5675-y 22252655

[B4] Cicero A. F. G. Fogacci F. Cincione I. (2021). Evaluating pharmacokinetics of bempedoic acid in the treatment of hypercholesterolemia. Expert Opin. Drug Metab. Toxicol. 17 (9), 1031–1038. 10.1080/17425255.2021.1951222 34197267

[B5] Corn G. Lund M. Andersson N. W. Dohlmann T. L. Hlatky M. A. Wohlfahrt J. (2024). Low-density lipoprotein cholesterol response to statins according to comorbidities and co-medications: a population-based study. Am. Heart J. 274, 102–112. 10.1016/j.ahj.2024.04.018 38710378

[B6] Elbe A. Foerster K. I. Blank A. Rose P. Burhenne J. Haefeli W. E. (2022). Evaluation of CYP2C19 activity using microdosed oral omeprazole in humans. Eur. J. Clin. Pharmacol. 78 (6), 975–987. 10.1007/s00228-022-03304-3 35238961 PMC9107402

[B7] EMA, (2024). Nilemdo Summary of Product Characteristics. Daiichi Sankyo europe GmbH.

[B8] EMA, (2020). Nustendi Summary of Product Characteristics. Daiichi Sankyo europe GmbH.

[B9] FDA (2019). Integrated Review NDA211616 Nexletol (bempedoic acid) tablets

[B10] FDA (2024). FDA’s examples of drugs that interact with CYP enzymes and transporter systems. Available online at: https://www.fda.gov/drugs/drug-interactions-labeling/healthcare-professionals-fdas-examples-drugs-interact-cyp-enzymes-and-transporter-systems. (Accessed February 21, 2025)

[B11] Halama B. Hohmann N. Burhenne J. Weiss J. Mikus G. Haefeli W. E. (2013). A nanogram dose of the CYP3A probe substrate midazolam to evaluate drug interactions. Clin. Pharmacol. Ther. 93 (6), 564–571. 10.1038/clpt.2013.27 23511711

[B12] Hiemke C. Bergemann N. Clement H. W. Conca A. Deckert J. Domschke K. (2018). Consensus guidelines for therapeutic drug monitoring in neuropsychopharmacology: update 2017. Pharmacopsychiatry 51 (1-02), e1. 10.1055/s-0037-1600991 29390205

[B13] ICH (2022). Bioanalytical method validation and sample analysis M10

[B14] Katzenmaier S. Markert C. Mikus G. (2010). Proposal of a new limited sampling strategy to predict CYP3A activity using a partial AUC of midazolam. Eur. J. Clin. Pharmacol. 66 (11), 1137–1141. 10.1007/s00228-010-0878-2 20680253

[B15] Mahmoudi M. Foerster K. I. Burhenne J. Weiss J. Mikus G. Haefeli W. E. (2021). Application of microdosed intravenous omeprazole to determine hepatic CYP2C19 activity. J. Clin. Pharmacol. 61 (6), 789–798. 10.1002/jcph.1789 33236774

[B16] Muhareb A. Blank A. Meid A. D. Foerster K. I. Stoll F. Burhenne J. (2023). CYP3A and CYP2C19 activity determined by microdosed probe drugs accurately predict voriconazole clearance in healthy adults. Clin. Pharmacokinet. 62 (9), 1305–1314. 10.1007/s40262-023-01287-7 37505445 PMC10450012

[B17] Nissen S. E. Lincoff A. M. Brennan D. Ray K. K. Mason D. Kastelein J. J. P. (2023). Bempedoic acid and cardiovascular outcomes in statin-intolerant patients. N. Engl. J. Med. 388 (15), 1353–1364. 10.1056/NEJMoa2215024 36876740

[B18] Ray K. K. Bays H. E. Catapano A. L. Lalwani N. D. Bloedon L. T. Sterling L. R. (2019). Safety and efficacy of bempedoic acid to reduce LDL cholesterol. N. Engl. J. Med. 380 (11), 1022–1032. 10.1056/NEJMoa1803917 30865796

[B19] Rohr B. S. Krohmer E. Foerster K. I. Burhenne J. Schulz M. Blank A. (2024). Time course of the interaction between oral short-term ritonavir therapy with three factor xa inhibitors and the activity of CYP2D6, CYP2C19, and CYP3A4 in healthy volunteers. Clin. Pharmacokinet. 63 (4), 469–481. 10.1007/s40262-024-01350-x 38393578 PMC11052790

[B20] SmPC (2022). “Plavix® 75 mg Filmtabletten,” Sanofi-aventis Deutschland.

[B21] Stoll F. Amato S. Sauter M. Burhenne J. Weiss J. Haefeli W. E. (2025). Effect of staggered vs. Simultaneous Co-administration of bempedoic acid on pharmacokinetics of pravastatin: randomized, cross-over clinical trial in healthy volunteers. Pharmaceutics 17 (1), 60. 10.3390/pharmaceutics17010060 39861708 PMC11768435

[B22] Stoll F. Blank A. Mikus G. Czock D. Weiss J. Meyer-Tönnies M. J. (2024). Evaluation of hydroxychloroquine as a perpetrator on cytochrome P450 (CYP) 3A and CYP2D6 activity with microdosed probe drugs in healthy volunteers. Eur. J. Drug Metab. Pharmacokinet. 49 (1), 101–109. 10.1007/s13318-023-00872-2 38114885 PMC10781839

[B23] Stoll F. Eidam A. Michael L. Bauer J. M. Haefeli W. E. (2022). Drug treatment of hypercholesterolemia in older adults: focus on newer agents. Drugs Aging 39 (4), 251–256. 10.1007/s40266-022-00928-z 35278206 PMC8995260

[B24] van der Heijden L. T. Opdam F. L. Beijnen J. H. Huitema A. D. R. (2024). The use of microdosing for *in vivo* phenotyping of cytochrome P450 enzymes: where do we stand? A narrative review. Eur. J. Drug Metab. Pharmacokinet. 49 (4), 407–418. 10.1007/s13318-024-00896-2 38689161 PMC11199305

[B25] Vay M. Meyer M. J. Blank A. Skopp G. Rose P. Tzvetkov M. V. (2020). Oral yohimbine as a new probe drug to predict CYP2D6 activity: results of a fixed-sequence phase I trial. Clin. Pharmacokinet. 59 (7), 927–939. 10.1007/s40262-020-00862-6 32060866 PMC7329762

[B26] Vay M. Sauter M. Mikus G. Burhenne J. (2019). Quantification of microdosed oral yohimbine and its major metabolite in human plasma in the picogram range. Bioanalysis 11 (16), 1459–1467. 10.4155/bio-2019-0129 31411489

